# A blended educational intervention program on Pap-test related behavior among Iranian women

**DOI:** 10.1186/s12978-021-01281-x

**Published:** 2021-11-13

**Authors:** Shahnaz Ghalavandi, Fatemeh Zarei, Alireza Heidarnia, Reza Beiranvand

**Affiliations:** 1 Department of Health Education and Health Promotion, Faculty of Medical Sciences, Tarbiat Modares University, P.O. Box: 14115-331, Tehran, Iran; 2 Department of Epidemiology and Biostatistics, School of Public Health, Tehran University of Medical Sciences, P.O. Box: 14115-331, Tehran, Iran

**Keywords:** Pap-test, Behavior, Health Education

## Abstract

**Objective:**

To assess the effect of a blended educational program to promote performing the PST among Iranian women.

**Design:**

In a randomized control trial four main variables; knowledge, attitude, self-efficacy, and practice about PST was evaluated using a man–made questionnaire for PST.

**Setting:**

Women aged 18–49 living in Andimeshk (Khuzestan, Iran), covered by 16 health centers, participated in study from November 2019 till April 2019.

**Method:**

The educational intervention conducted to increasing women’s performing the PST. The experimental group received an intervention, whereas the control group received usual care. Participants were tested at four-time points: pre-test (baseline), post-test 1 (immediately after the program’s completion) post-test 2 (4 weeks after the program’s completion) and post-test 3 (12 weeks after the program completion).

**Results:**

A total of 84 women with average aged 32.27 (42 in the experimental group, 42 in the control group) were recruited from 16 health centers in Andimeshk, southern Iran. Significant group differences were found at different times in knowledge, attitude, self-efficacy, and practice about PST.

**Conclusion:**

A blended method was effective in sustaining the effects of the educational program in the experimental group. The development of appropriate teaching method on restricted situation such as COVID-19 pandemic to promote participation is suggested.

## Plain Language Summary

Cervical cancer is the fourth most common cancer among women. The Pap-test is considered the most economical and efficient method for screening cervical cancer and is a simple method without any side effects. Given that awareness with proper methods regarding the initial symptoms, timely diagnosis and treatment are vital for controlling cancer.

In this study, we assessed the effect of a blended educational program to promote performing Pap-test among 84 Iranian women. Therefore, a study to assess four main variables; knowledge, attitude, self-efficacy, and practice about PST was designed. We asked women aged 18–49 living in Andimeshk (Khuzestan, Iran) covered by 16 health centers. The educational program to increase women’s performance in the PST using online technology regarding participants' convenience, accessibility, and availability of educational content. Women were divided into two groups. One group received an educational program, the second group remained in control and did not receive any education. All women were asked for their knowledge, attitude, self-efficacy and practice of PST three times immediately, 4 and 12 weeks after one-month training.

Our assessment showed significant group differences at different times in knowledge, attitude, self-efficacy, and practice about PST.

In conclusion, effective online education in sustaining the effects of the educational program in the experimental group happened. Moreover, we experienced an appropriate teaching method for restricted situations such as the COVID-19 pandemic to promote educational participation.

## Introduction

Cervical cancer is the fourth most common cancer among women. In 2018, 570 thousand new cases of cervical cancer occur globally [1], of which over 85% occur in less developed nations [2]. Based on WHO’s report, in 2018, cervical cancer came second after breast cancer in terms of incidence in Asia and the Middle East’s cancer ranking [3]. In Iran, its incidence is 4.5 per 100,000 persons. Annually, of every 123 women, one is affected with cervical cancer, and of every 100,000 women, nine die of this cancer [4]. The WHO has estimated that by 2030, this cancer will be the cause of death of approximately 474,000 women per year and 95% of these deaths will occur in low- and middle- income countries [5].

The most important risk factors of this disease are, pregnancy at a young age, multiple sexual partners, human immunodeficiency virus (HIV), herpes simplex virus (HSV), cytomegalovirus (CMV), HPV, sexually transmitted infections (STI), weakness of the body’s immune system, genetic factors and exposure to certain chemicals [6]. Cervical cancer is known as a preventable cancer, as the duration of the pre-cancerous stage is lengthy. However, there is rapid access to trustworthy screening programs, and the primary lesions can be treated on time [4, 5]. The stage of the disease at diagnosis has direct impact on improving prognosis in patients. During the early stages of the disease the five-year survival rate is 92%, whereas, in its late stages it is 13% [4]. The incidence, morbidity & mortality rates of cervical cancer have declined in most developed nations because of the routine tests and Pap smear testing.

As a rule, preventive health behaviors lead to positive health effects. The Pap smear test (PST) is a cervical screening test used to detect potentially pre-cancerous or cancerous processes in the cervix. Cervical cancer can remain in the non-invasive stage for over 20 years and its abnormal cells are detectable with PST. In cases of timely detected and treated cases, PST can prevent the abnormal growth of lesions by 40–60% [7].

Cervical cancer screening (Pap smear testing) identifies pre-cancerous lesions at various stages so they can easily be treated. In developing countries, access to these types of preventive measures is limited, and cervical cancer is usually left undiagnosed until it reaches its advanced stages [2]. Despite the considerable success of the PST in detecting cervical cancer, in developing countries the rate of participation is 5% only. In high-income countries like the US however, this rate is approximately 90%. Many studies in Iran have reported the low participation rate of this test. For example, this rate has been reported at 27.1% in Babazadeh et al.’s study [8], 50% in Farzaneh et al.’s study [9], and 58.6% in Ghalavandi et al.’s research in Andimeshk city [10].

PST is considered as the most economic and efficient method for screening cervical cancer and as a simple method without any side effects [11]. If performed correctly and with the right instruments the PST can detect cervical cancer with a specificity of 70–95% [12]. Based on literature, 60% of cervical cancer related deaths are preventable by PST screening [5].

Two interwoven steps in health education planning are the utilization of appropriate theories and method. Recent literature in Iran indicates that women do not take the PST very seriously [13, 14]. Nevertheless, knowledge, attitude and self-efficacy are probably the main three constructs that affect women’s practice regarding the PST. In other words, earlier literature indicates that improving the educational status not only increases pap smear knowledge, but is also associated with women’s attitude and self-efficacy regarding PST [15, 16]. Moreover, by strengthening positive attitude and raising self-efficacy in women, the intention to perform the PST is also reinforced [10, 17, 18]. The individual must also have perceived self-efficacy to perform the PST, i.e., she should believe that she will be able to routinely perform the PST [14, 15]. Promoting Pap smear-related behaviors with an appropriate educational method is important. Individual and social enabling factors such as literacy level, age, socioeconomic status, as well as access limiting situation such as COVID-pandemic and lockdown affect educational method selection. Therefore, choosing the proper training method is as important as promoting behavior. Blended learning, facilitates educational program due to providing training both in online and offline ways. Given that awareness regarding the initial symptoms, timely diagnosis and treatment are all vital for controlling cancer, and that literature shows that the PST is considerably performed less in Iran than in other countries, there is a firm need towards an educational intervention [19]. Hence, the current study was conducted to determine the impacts of the educational intervention on performing the PST among women living in Iran’s Andimeshk city.

## Methods and materials

### Design

Women living in Andimeshk (Khuzestan, Iran), covered by 16 health centers, participated in a randomized controlled trial [IRCT20191206045626N1] study from November 2019 till April 2019.

### Participants/inclusion, and exclusion criteria

Participants in this study were women aged 18–49 attending comprehensive health service centers in Andimeshk and were stratified randomly. Inclusion and exclusion criteria included women of reproductive age (who had been married at least once, regardless of their current marital status), residents of Andimeshk, literate, 18–49-years-old, with poor and average knowledge mean scores (10). Participants with a history of cervical cancer or other sexually transmitted diseases and unwillingness to continue were excluded from the study.

### Sampling

Andimeshk city has 16 comprehensive health centers. Sampling was done based on low and average knowledge mean scores of women assigned to each class by simple random sampling. Each of the centers was considered a class through stratified random sampling. 84 of those whose total knowledge score (based on the descriptive phase of the study) (10) was poor and/or average (poor: 0–4, average: 4–8, good: 8–13) were selected. Women possessing the inclusion criteria who had active profiles in the health centers were contacted. The 84 participants were randomly through Random-ize.com and equally assigned to the experimental and control groups (42 in each). Data gathering in each group was continued until it reached the desired sample size.

### Assessment tool

The assessment tool was a man-made questionnaire for PST screening. It contained 5 sections; the first section consisted of demographic questions (Table 1). The knowledge domain had 13 questions with three-item responses (correct, incorrect, do not know), such that ‘correct’ answers garnered a score of 1 and ‘incorrect’ and ‘do not know’ garnered a score of zero. The attitude domain contained 12 questions with five-point Likert type responses (completely uncertain, somewhat uncertain, no idea, somewhat certain, completely certain). In the attitude and self-efficacy domains, the questions garnered scores between 1 and 5 depending on the responses. The practice domain comprised 4 two-choice questions (yes, no) that would score 1 or 0 depending on the answer. The self-efficacy domain of this questionnaire is based on the questionnaire used by Fernandez et al. among Mexican–American women in 2009 (20). To use this questionnaire, it was translated from English into Persian and its validity and reliability were examined. To this end, we took permission from its creators and after obtaining the guideline the forward–backward translation was done. Two experts from the health domain separately translated the scales into Persian. After an accurate review and cultural adaptation minor modifications were made to the questionnaire. The two translated versions were then compared with each other and the translated items were adapted to each other. Thereafter, the final version was back-translated into English to compare and adapt it to the original version. Content validity of the domains (knowledge, attitude, practice, and self-efficacy toward PST) was determined through an expert panel. The face validity was defined via the impact score and lay people’s opinions. The reliability of the questionnaire was determined by the test–retest method (intra-class correlation coefficient) and internal correlation (calculating the Alpha-Cronbach coefficient). Cronbach’s alpha coefficient was used to measure the internal correlation, which was 0.81 in the awareness domain, 0.79 in the attitude domain, 0.85 in the self-efficacy domain and 0.91 in the practice domain. The final version of the questionnaire contained 44 items.

**Table 1:** Basic characteristics of participants in the intervention and control groups

Variables		Intervention group (n = 42)	Control group (n = 42)
		Frequency (%)^a^	
Family’s economic status	Weak	3 (7.1)	6 (14.3)
	Moderate	27 (64.3)	24 (57.1)
	Good	12 (28.6)	12 (28.6)
Level of education	Elementary	2 (4.8)	1 (2.4)
	Intermediate	20 (47.6)	25 (59.5)
	High school diploma	11 (26.2)	15 (35.7)
	Bachelor and above	9 (21.4)	1 (2.4)
Employment status	Housewife	31 (73.8)	39 (92.9)
	Freelancer	2 (4.8)	0 (0)
	Employment	9 (21.4)	3 (7.1)
Husband’s employment status	Freelancer	12 (28.6)	7 (16.7)
	Employment	30 (71.4)	35 (83.3)
Previous Pap testing	Yes	16 (38.1)	20 (47.6)
	No	26 (61.9)	22 (52.4)

	Mean ± SD^b^	
Age	31.83 ± 6.62	32.71 ± 7.41
Age at first intercourse	22.79 ± 3.33	22.33 ± 4.13

^a^Chi-Square test

^b^Independent samples T-Test

### Intervention development

We developed the contents of the intervention using the blended learning method. Through a series of focus group discussions with participants and health professionals, we determined that the best format during the COVID-19 pandemic period would be to deliver the 7-day course over a period of four weeks, both multimedia-message-based and online discussion would be delivered via smartphones. Directed by the four main goals of the program as cervical cancer awareness, this program was abbreviated as *SADRA*. *Sadra (سدرا) is a term we have coined for a woman who is aware of cervical cancer prevention.*

The smartphone multimedia messaging intervention identifies knowledge regarding cervical cancer and PST screening; attitudes (myths and misunderstandings) related to PST; how, when, and why a married woman needs to plan for a PST; and triggers off a health behavior, such as setting an appointment for a PST examination.

The content of the *SADRA* intervention was not individually tailored to each participant, as all participants had the same level of knowledge, as mentioned in the inclusion criteria. Furthermore, they were homogenic in terms of socio-cultural status. Therefore, the educational contents generally included:

(1) Basic health information about the cervix and cervical cancer, including statistical facts, such as cervical cancer incidence and mortality among Iranian women, particularly women from Khuzestan province compared to women from other parts of Iran.; (2) introducing the PST as a screening method for early diagnosis of cervical cancer; (3) information about accessibility and availability of local healthcare centers; (4) the cost of PST and information on how to plan for the test’s health insurance; (5) stories that described Iranian women overcoming socio-cultural barriers to do the PST; (6) who the PST will be performed by a midwife (7) statements of women who have done the PST.

The *SADRA* intervention was designed as a blended education; an online educational class and offline educational material in the form of multimedia. It contained four weeks of online classes as well as multimedia message delivery via the smartphone. Participants selected their preferred time to be online for the online discussion and received multimedia messages. Accordingly, each Thursday at 9:00 P.M, four 30-min online class discussions were moderated by two mentors identified as a health professional and a midwife affiliated with health centers in Andimeshk. These classes were held on ‘BigBlueButton’, an online teaching platform provided by Tarbiat Modares University. Participants also agreed to receive multimedia messages each day for four weeks at 10:00 a.m. Participants would receive a reminder notification for the online class through their social media (such as WhatsApp and Telegram) an hour before the online class would begin. To enhance participants’ adherence to the *SADRA* intervention program, we bought internet service for their smartphones.

The *SADRA* intervention involves a high level of interaction through quizzes and questions. The 29 questions and quizzes delivered during the four-week intervention asked participants about their knowledge on cervical cancer mortality, and PST procedures and readiness. At the end of the quizzes the online health educators would address the participants’ concerns and questions. On the last day of the intervention, we tested their knowledge to ascertain whether they had learnt the SADRA intervention. We also provided them with information about health centers near them. No special training was delivered to the control group during the 4 weeks of the intervention.

### Measures

We conducted a face-to-face interview for pre-testing at each participant’s preferred place using a paper-and-pencil questionnaire. Since the post-test and follow-up testing coincided with the COVID-19 epidemic in Iran, an online questionnaire was used. We collected information from participants at 3 different time points: study enrollment (baseline); one month after completing the educational intervention (post-test); and 12 weeks after completing the study (follow-up test).

#### Outcome measures

The primary outcomes of interest included: (1) changes in knowledge, attitudes, and beliefs about cervical cancer screening and the PST; (2) intention to undergo Pap-testing.

#### Baseline measures

We collected participants’ sociodemographic information (age, marital status, education, income, husband’s job and employment status), health-related information (Pap-testing history). We also collected information about knowledge and attitudes related to the PST, cultural health beliefs and attitudes, self-efficacy regarding PST and screening, and intention to do the PST as part of cervical cancer prevention. For knowledge, attitude, and practice we used a man-made 29-item questionnaire. For measuring self-efficacy regarding cervical cancer prevention and screening, we used the 8-item scale by Fernández et al. [20]. The psychometric properties of each domain/scale in the pre- and post-tests are reported in the Appendix.

#### Post-test measures

At one-month post-intervention, we administered a post-test survey of the same items we collected at baseline. At the 12 weeks online follow-up, we asked participants if they had done the PST and, if they had not, their reasons for that decision.

### Data analysis

STATA-16 software was used for data analysis. To measure the difference between the two test and control groups in the pre-intervention stage, in terms of family economic status, level of education, employment status, husband’s employment status, history of PST, chi-square test and to measure the difference between the two groups in terms of employment’ status, Fisher’s exact test was used.

Also, at this stage, the independent samples t-test was used to measure the difference between the two groups in terms of mean age and mean age at first intercourse; the error level was set at 0.05. Repeated Measures and linear regression were used to assess the effect of education on the mean scores of knowledge, attitude, self-efficacy, and practice at baseline, immediately, 4 weeks and 12 weeks after the intervention.

To evaluate the effect of the educational intervention on the mean scores of knowledge, attitude, self-efficacy, and practice during the study in each group separately, the statistical test of repeated measures was used. The Greenhouse–Geisser approach was used if Mauchly’s test result was significant and the sphericity assumption was applied if Mauchly’s test was not significant.

Linear regression was used to measure the differences between the two groups in the mean scores of knowledge, attitude, self-efficacy, and practice at each stage. Furthermore, in analyzing the mean scores of outcome variables (knowledge, attitude, self-efficacy and practice) in each of the stages between the two groups, to avoid confusion, the mean of the previous stage was adjusted. Thus, in analyzing the stage immediately after the intervention, the stage before the intervention; in analyzing the 4 weeks post-intervention stage, the mean of the immediate post-intervention stage, and in analyzing the 12 weeks post-intervention stage, the mean of the 4 weeks post-intervention stage was adjusted. It should be noted that the analyzes were performed with the intention to treat (ITT) approach at an error level of 0.05.

## Consort

### Results

#### Participants’ characteristics

Table 1 shows characteristics of women in the intervention and control groups, which were mostly similar. Most women were with a mean age of 31.83 ± 6.62 and 32.71 ± 7.41 years in the intervention and control groups, respectively. The majority in both groups were housewives and of moderate economic status. They had undergone at least one PST (Table 1).

#### Comparison of mean PST knowledge, attitude, practice, and self-efficacy scores between women in intervention and control groups

The total number of participants dropped from 84 at baseline to 83 at the first evaluation.

#### Knowledge about PST

The mean knowledge scores in the experimental and control groups before the study were 5.33 ± 1.76 and 4.90 ± 1.91, respectively, but this difference was not significant (P = 0.285). 12 weeks after the intervention the mean knowledge scores in the experimental and control groups increased to 11 ± 1.18 and 6.02 ± 1.31, respectively (P < 0.001) (Table 2). Immediately, 4 and 12 weeks after the intervention the mean knowledge scores in the experimental group were significantly higher than those in the control group (P ˂ 0.001) (Table 3).

**Table 2:** Evaluation of the change in mean Pap smear knowledge, attitude, self-efficacy, and practice scores of women participating in^a^ the study, by groups

Participants		Baseline	Immediately after	4 weeks after	12 weeks after	P-value^b^
Intervention	Knowledge	5.33 ± 1.76	10 ± 1.32	10.55 ± 1.10	11 ± 1.18	< 0.001
	Attitude	28.14 ± 6.52	37.24 ± 7.62	38.05 ± 7.55	39.29 ± 7.17	< 0.001
	Self-efficacy	18.67 ± 6.26	26.16 ± 5.46	28.19 ± 4.73	29.12 ± 4.44	< 0.001
	Practice	2.98 ± 1	3.33 ± 0.816	3.45 ± 0.670	3.88 ± 0.328	< 0.001
Control	Knowledge	4.90 ± 1.91	5.93 ± 1.86	6.10 ± 1.76	6.02 ± 1.31	< 0.001
	Attitude	29.10 ± 6.95	29.26 ± 5.57	28.83 ± 5.54	28.57 ± 5.46	0.480
	Self-efficacy	19.86 ± 6.43	20 ± 5.88	20.02 ± 5.25	19.88 ± 4.95	0.927
	Practice	2.64 ± 1.22	2.88 ± 1.19	2.88 ± 1.19	2.93 ± 1.11	0.144

^a^Before the intervention, immediately, 4 weeks and 12 weeks after the intervention

^b^Greenhouse–Geisser Repeated Measures

**Table 3:** The effect of the intervention on mean Pap smear knowledge, attitude, self-efficacy, and practice scores of participants immediately, 4 weeks, and 12 weeks after the intervention

Variables		Baseline		Immediately after		4 weeks after		12 weeks after	
		Coefficients	P-value^a^	Coefficients^b^	P-value^a^	Coefficients^c^	P-value^a^	Coefficients^d^	P-value^a^
Knowledge	Control	4.90	< 0.001	3.43	< 0.001	1.34	< 0.001	2.94	< 0.001
	Intervention	0.429	0.285	3.85	< 0.001	1.18	< 0.001	2.72	< 0.001
Attitude	Control	29.09	< 0.001	9.76	< 0.001	0.655	0.423	2.53	0.03
	Intervention	− 0.952	0.519	8.61	< 0.001	1.53	< 0.001	2.33	< 0.001
Self-efficacy	Control	19.85	< 0.001	6.50	< 0.001	4.12	0.423	2.81	0.03
	Intervention	− 1.19	0.393	6.99	< 0.001	3.24	< 0.001	2.27	< 0.001
Practice	Control	2.64	< 0.001	0.88	< 0.001	0.325	0.007	1.34	< 0.001
	Intervention	0.333	0.176	0.20	0.120	0.170	0.028	0.61	< 0.001

^a^Linear regression at α = 0.05

^b^Adjusted for mean scores before the intervention

^c^Adjusted for mean scores immediately after the intervention

^d^Adjusted for mean scores 4 weeks after the intervention

#### Attitudes about PST

The mean attitude scores in the experimental and control groups before the study were 28.14 ± 6.52 and 29.10 ± 6.95, respectively, but this difference was not significant (P = 0.519). 12 weeks after the intervention, the mean attitude score in the experimental group had increased to 39.29 ± 7.17 (P ˂ 0.001) (Table 2). However, it had not increased significantly in the control group (P = 0.480) (Table 2). Immediately, 4 weeks, and 12 weeks after the intervention, the mean attitude scores in the experimental group were significantly higher than those in the control group (P < 0.001) (Table 3).

#### Self-efficacy about PST

The mean self-efficacy scores in the experimental and control groups before the intervention were 18.67 ± 6.26 and 19.86 ± 6.43, respectively, but this difference was not significant (P = 0.393). 12 weeks after the intervention, the mean self-efficacy score in the experimental group had increased to 29.12 ± 4.44 (P < 0.001) (Table 2). However, it had not increased significantly in the control group (P = 0.927) (Table 2). Immediately, 4 weeks, and 12 weeks after the intervention, the mean self-efficacy scores in the experimental group were significantly higher than those in the control group (P < 0.001) (Table 3).

#### Practice about PST

The mean practice scores in the experimental and control groups before the intervention were 2.98 ± 1 and 2.64 ± 1.22 (P = 0.144), respectively. 12 weeks after the intervention the mean practice score in the experimental group increased to 3.88 ± 0.328 (P < 0.001) (Table 2). Comparing the mean practice scores in the experimental and control groups, no significant difference was observed immediately and 4 weeks after the intervention (P > 0.05), while 12 weeks after the intervention there was a significant difference between the two groups’ mean practice scores (P < 0.001) (Table 3).

## Discussion and conclusion

### Discussion

Based on our results, a statistically significant difference was observed between the two groups in terms of the mean scores of knowledge, attitude, and self-efficacy immediately, 4 weeks, and 12 weeks after the intervention. This statistical difference was observed in the mean practice scores of the two groups 12 weeks later too.

The mean knowledge scores showed no statistically significant difference between the two groups before the intervention. However, significant differences were observed between the pre-intervention score and those immediately, 4 and 12 weeks after the intervention in the test group (P < 0.001). The mean knowledge scores significantly differed before and immediately, 4 and 12 weeks after the intervention in the control group too (P < 0.001), which may be due to the participants’ increased interest in the subject matter and their personal studies on it. Upon comparing the two groups, the mean knowledge scores in the test group immediately after the intervention (adjusted for the pre-intervention knowledge score), 4 weeks after the intervention (adjusted for the immediate post-intervention knowledge score), and 12 weeks after the intervention (adjusted for the knowledge score 4 weeks post-intervention) were higher than the knowledge scores in the control group at the same stages (3.85, 1.18 and 2.72, respectively) and statistically significant (P < 0.001). This means that although the knowledge score significantly increased in both the control and test groups at all the stages following the intervention, the overall comparison of the two groups showed that this increase was significantly higher in the test group. This hints at the impact of the educational intervention on raising the knowledge score, which is consistent with the findings of other studies [19, 21–27].

The pre-intervention mean attitude scores did not show any statistical difference between the test and control groups. However, it did show statistically significant differences with the mean scores immediately, 4 and 12 weeks after the intervention in the test group (P < 0.001). On the other hand, the pre-intervention mean attitude score in the control group did not statistically differ with the mean scores at the three post-intervention time-points (P = 1). Upon comparing the two groups, the mean attitude scores in the test group immediately after the intervention (adjusted for the pre-intervention attitude score), 4 weeks after the intervention (adjusted for the immediate post-intervention attitude score), and 12 weeks after the intervention (adjusted for the attitude score 4 weeks post-intervention) were higher than the attitude scores in the control group at the same stages (8.61, 1.53 and 2.33, respectively) and statistically significant (P < 0.001). These results indicate the impact of the intervention on improving the attitude score, and are consistent with those of similar studies [21, 23, 24, 28]. Roland et al. [29] showed that the educational intervention had no impact on raising the participants’ attitude score, a finding contrary to ours. This inconsistency may be attributed to the lack of dynamicity of the educational method, which used documented material at the beginning of the study, whereas, in our study, the educational material was presented in consecutive sessions where in the participants were in touch with the educator and there were Q & A sessions as well.

The pre-intervention mean self-efficacy scores did not show any statistical difference between the test and control groups. However, they did show statistically significant differences with the mean scores immediately, 4 and 12 weeks after the intervention in the test group (P < 0.001). On the other hand, the pre-intervention mean self-efficacy score in the control group did not statistically differ with the mean scores at the three post-intervention time-points (P = 1). Upon comparing the two groups, the mean self-efficacy scores in the test group immediately after the intervention (adjusted for the pre-intervention self-efficacy score), a four weeks after the intervention (adjusted for the immediate post-intervention self-efficacy score), and 12 weeks after the intervention (adjusted for the self-efficacy score 4 weeks post-intervention) were higher than the self-efficacy scores in the control group at the same stages (6.99, 3.24 and 2.27, respectively) and statistically significant (P < 0.001). These results were consistent with those of similar studies [19, 30, 31].

The pre-intervention mean practice score did not statistically differ between the test and control groups. The mean practice score in the test group significantly differed between pre-intervention and the three post-intervention time-points (P < 0.001). However, in the control group the mean pre-intervention practice score did not statistically differ with those after the intervention (P > 0.05). Comparison of the practice scores of the two groups indicated that a much longer time is required to change women’s practice regarding the PST as compared to the time required to increase knowledge, attitude, and self-efficacy. Our results indicated the positive effect of education on raising the practice score. Our results are consistent with those of studies that have assessed practice change at least 8 weeks after the intervention [19, 27, 30, 32]. A systematic review study conducted by Ghareh-naz et al. [33] on studies conducted between 2000 and 2017 also showed that health education regarding cervical cancer and screening with PST improved women’s practice in performing the PST.

Waldez et al. [29] observed that education did not cause a significant difference in the participants’ practice, which is contrary to our findings. It appears that the reasons of inconsistency between our findings and theirs are that they had chosen their samples from low-income women, and the training took place in a single session and was not continuous. On the other hand, in our study, the participants had been selected randomly from all economic levels, and the training took place continuously for an entire month.

Although these findings support the effectiveness of the educational intervention, this study had some limitations including the effects of other information sources on the experimental and control groups. Moreover, Sociocultural and personal barriers to cervical cancer prevention and screening as well as COVID pandemic was the other points that overshadowed our educational program. However, this research had some strengths too, that using the blended learning method made educational intervention was easy in accessible for participating educational program in COVID pandemic period.

## Conclusion

Health educational program improved knowledge, attitude, and performance of the women under study as for doing PST. Therefore, designing for and implementing an educational interventions program using online technology regarding participants convenience for accessibility and availability of educational contents is recommended”. Conducting similar studies using virtual platform to make accessible educational material among restricted situation such as COVID pandemic is recommended.

## Data Availability

The original data from the survey is available in a Spss spreadsheet.

## Abbreviations

HIV: Human immunodeficiency virus
HSV: Herpes simplex virus
CMV: Cytomegalovirus
HPV: Human papilloma virus
STI: Sexually transmitted infections
PST: Pap-smear test
ITT: Intention to treat
